# White matter tract changes in pediatric posterior fossa brain tumor survivors after surgery and chemotherapy

**DOI:** 10.3389/fnimg.2022.845609

**Published:** 2022-09-20

**Authors:** Jeffrey Tanedo, Niharika Gajawelli, Sharon Guo, Mary Baron Nelson, Natasha Lepore

**Affiliations:** ^1^CIBORG Laboratory, Department of Radiology, Children's Hospital Los Angeles, Los Angeles, CA, United States; ^2^Department of Biomedical Engineering, University of Southern California, Los Angeles, CA, United States; ^3^Keck School of Medicine, University of Southern California, Los Angeles, CA, United States

**Keywords:** posterior fossa tumors, diffusion tensor (DT) MRI, white matter (WM), long term adverse effects, tract specific analysis

## Abstract

**Background:**

Survivors of pediatric posterior fossa brain tumors are susceptible to the adverse effects of treatment as they grow into adulthood. While the exact neurobiological mechanisms of these outcomes are not yet understood, the effects of treatment on white matter (WM) tracts in the brain can be visualized using diffusion tensor (DT) imaging. We investigated these WM microstructural differences using the statistical method tract-specific analysis (TSA). We applied TSA to the DT images of 25 children with a history of posterior fossa tumor (15 treated with surgery, 10 treated with surgery and chemotherapy) along with 21 healthy controls. Between these 3 groups, we examined differences in the most used DTI metric, fractional anisotropy (FA), in 11 major brain WM tracts.

**Results:**

Lower FA was found in the splenium of the corpus callosum (CC), the bilateral corticospinal tract (CST), the right inferior frontal occipital fasciculus (IFOF) and the left uncinate fasciculus (UF) in children with brain tumors as compared to healthy controls. Lower FA, an indicator of microstructural damage to WM, was observed in 4 of the 11 WM tracts examined in both groups of children with a history of posterior fossa tumor, with an additional tract unique to children who received surgery and chemotherapy (left UF).

**Conclusions:**

Our findings indicate that a history of tumor in the posterior fossa and surgical resection may have effects on the WM in other parts of the brain.

## Introduction

Central nervous system (CNS) tumors are second in frequency only to leukemia among cancers affecting children, but are still the most common cause of cancer death (Udaka, 2018) in children ages 0–14 years in the United States, with an incidence rate of approximately 5.83 per 100,000 person-years (Ostrom et al., 2017). Of these tumors in children, over half are in the posterior fossa, making it the most common location for CNS tumors. Treatment for these tumors can include a combination of surgical resection, chemotherapy, and cranial or craniospinal irradiation. New developments in these therapies, earlier detection (Duc et al., 2020), and improved post-treatment monitoring have increased the survival rates for pediatric patients. However, children are most susceptible to the adverse effects of treatment during this period of significant brain development (Macartney et al., 2014). Thus, the urgency to examine the long-term adverse outcomes of these treatments has also increased.

The exact neurobiological mechanisms leading to adverse outcomes from these therapies are not yet understood. However, the effects on brain anatomy may be visualized, quantified, and analyzed through magnetic resonance imaging (MRI) (Kim et al., 2008; Ikonomidou, 2018; Jacob et al., 2018; Duc, 2020). For example, global reductions in both gray and white matter (WM) volumes have been observed and correlated with neurocognitive decline after treatment (Ailion et al., 2017).

WM is important in mediating the functional connectivity for many neurobehavioral operations (Filley and Fields, 2016) and is susceptible to damage from radiation and chemotherapy. Thus, particular attention has been given to WM tract alterations with diffusion-weighted imaging (DWI). DWI is an MRI method which captures the diffusion of water molecules through brain tissues and is particularly useful in analyzing WM tracts. Healthy WM typically consists of bundles of myelinated axons organized into tracts, which connect different parts of the brain. The restricted diffusion of water molecules through these bundles can be characterized through different metrics, the most popular of which is called fractional anisotropy (FA). FA describes the degree of deviation from purely isotropic Brownian motion of water molecules in the brain, where higher FA values indicate highly anisotropic random motion (Basser et al., 1994).

Several studies have demonstrated lower FA in the WM tracts of children with brain tumors after receiving variable combinations of the three common treatments, surgery, chemotherapy, and radiation, compared to healthy controls, which may indicate WM damage due to treatment. These lower FA values have been theorized to indicate less restricted diffusion of water molecules and furthermore theorized to represent a loss of microstructural integrity, reduced bundle organization or axonal damage (Scholz et al., 2014). Before either chemotherapy or radiation, surgical resection of posterior fossa tumors alone impacts the supratentorial brain, as evidenced by lower FA and decreases to WM volume in structures such as the corpus callosum and corona radiata (Reddick et al., 2005; Rueckriegel et al., 2010; Glass et al., 2017). The combination of surgery, chemotherapy and radiation has been shown to be detrimental, with many studies documenting reduced FA in several brain structures, including the corpus callosum and frontal WM (Fouladi et al., 2004; Reddick et al., 2005; Monje et al., 2007; Jacola et al., 2014). Although literature solely focused on chemotherapy's effects on children with brain tumors is sparse, our recent paper in Baron Nelson et al. (2021) found patterns of FA differences in gray and white matter structures associated with the effects of surgery and chemotherapy compared to surgery alone through a whole brain voxel-wise analysis. Thus, examining differences in FA values is important in determining WM damage in relation to different treatments.

Many approaches have been developed to identify the FA values in WM tracts. Manually drawn regions of interest (ROIs) ensure anatomical accuracy. However, manual methods like this have been replaced by the development of automated methods of WM identification as the number of subjects in studies has increased. To address the increasing workload and time required to analyze larger datasets, tract-based spatial statistics (TBSS) (Smith et al., 2006) became the standard automated method in the study of WM tracts. TBSS accomplishes this by first non-linearly registering the FA maps from individual scans. The normalized FA maps are averaged and eroded to produce a WM skeleton which represents the core of all the WM tracts common to the initial scans. With the method's rise in popularity, researchers have become increasingly aware of its limitations. For example, TBSS's projection onto an entire WM skeleton does not allow a researcher to distinguish between distinct but adjacent WM tracts (Bach et al., 2014).

Tract specific analysis (TSA) was designed to remedy these problems by segmenting individual WM tracts onto population specific templates (Yushkevich et al., 2009; Zhang et al., 2010). Tensors are then projected onto a medial sheet which both defines the skeleton and informs the boundary of a WM tract. The maximum or mean tensor values can be calculated along a spoke extending perpendicularly from a point on the medial sheet to the tract boundary. From these values, DTI metrics such as FA can be calculated. Thus, TSA can provide FA values for specific WM tracts while reducing noise from adjacent tracts. A previous study from this lab comparing TBSS and TSA has shown that in a comparison between a congenital blind group and healthy sighted controls, TSA shows greater sensitivity compared to TBSS in detecting subtle differences in WM (Lao et al., 2015).

In this study, we investigate WM microstructure differences between three groups: (1) pediatric brain tumor survivors who underwent surgery only, (2) those who underwent surgery and received chemotherapy, and (3) healthy controls, by utilizing TSA to compare FA values across 11 major WM tracts. We seek to further delineate neuroimaging findings from our previous study on the same cohort, which identified clusters of lower FA in children treated with both surgery and chemotherapy than in those treated with surgery in the superior longitudinal fasciculus (SLF) bilaterally and in the left uncinate fasciculus (UF) using whole supratentorial brain voxel-wise analysis of FA (Baron Nelson et al., 2021). In that study, we demonstrated clusters wherein the Z-score in the difference between the two groups being measured was greater than 2 standard deviations. These contiguous clusters may span several known white matter tracts and gray matter structures. The current study zooms into 11 specific white matter structures and gray matter structures. This allows us to localize clusters more precisely on particular tracts, including white matter tracts which were correlated with the location of significant clusters found in the previous study. Based on our previous study and the findings of others, we hypothesize that TSA will indicate a pattern of injury to the corpus callosum, SLF, and UF in children with brain tumors compared to healthy controls, and that those children treated with chemotherapy in addition to surgery will have a pattern of injury that is more widespread than children treated with surgery alone.

## Materials and methods

### Participants

Participant demographics and recruitment are the same as a prior study (Baron Nelson et al., 2021). All included participants were between 6 and 17 years old, inclusive, and were also required to speak and read either English or Spanish. All included patient participants had: (1) tumor location in the posterior fossa – cerebellum or fourth ventricle, (2) complete tumor resection with no evidence of metastasis more than 1 year after treatment, and (3) at least 1 year since the patient's last treatment for brain tumor. All included control participants had no prior history of traumatic brain injury or neurological disease. Potential participants were excluded if they met the following criteria: (1) metal in the body, (2) preterm birth, (3) neurodevelopmental disability, (4) traumatic brain injury, or (5) turning 18 years old during study duration. Potential patient participants were excluded if they had a recurrent tumor, residual disease outside of the posterior fossa or a history of posterior fossa syndrome. Potential control participants were also excluded if they needed sedation for an MRI scan. Patient demographics such as age, gender, race/ethnicity, tumor diagnosis site and time off treatment can be found in Table 1.

**Table 1:** Patient demographics by treatment group compared to controls.

**Variable**		**Treatment group**			***p*-value**
		**Surgery (*n* = 15)**	**Surgery+Chemo** **(*n* = 10)**	**HC (*n* = 21)**	
Age – mean (SD)		10.60 (4.12)	12.50 (3.54)	10.52 (2.27)	0.26^a^
Age at diagnosis – mean (SD)		5.55 (3.08)	3.77 (3.36)	NA	0.19^a^
Sex	Male	4 (27%)	6 (60%)	13 (62%)	0.09^b^
	Female	11 (73%)	4 (40%)	8 (38%)	
Patient Race/ethnicity	White Non-Hispanic Hispanic/Latino	13 (87%)	9 (90%)	18 (86%)	Race – 0.84^b^ Ethnicity – 0.23^b^
		6 (46%)	3 (33%)	10 (56%)	
		7 (54%)	6 (67%)	8 (44%)	
	Black/African American	1 (7%)	0 (0%)	0 (0%)	
	Asian	1 (7%)	1 (10%)	3 (14%)	
Diagnosis	Medulloblastoma	0 (0%)	6 (60%)	NA	**0.0005** ^b^
	Ependymoma	0 (0%)	1 (10%)		
	Astrocytoma	2 (14%)	0 (0%)		
	Pilocytic Astrocytoma	12 (80%)	2 (20%)		
	Other	1 (6%)	1 (10%)		
Time off treatment (years) – mean (SD)		4.67 (3.29)	8.18 (4.97)	NA	**0.04** ^a^

**Bold font** indicates significant *p*-value (*p* ≤ 0.05).

^a^two-sided t-test performed in STATA.

^b^chi-square test performed in STATA.

The Institutional Review Board at Children's Hospital Los Angeles approved this study. The Neuro-oncology database and clinic lists were used to identify potential subjects. Recruitment of subject families took place in clinic or by mail or phone.

Eight of 10 children (80%) in the chemotherapy group received intensive marrow-ablative chemotherapy followed by autologous hematopoietic stem cell transplant (AuHSCT) with some combination of thiotepa, etoposide or carboplatin as the conditioning regimen. Most children in the chemotherapy group also received cisplatin, cyclophosphomide, and vincristine.

Two subject participants from the surgery group were excluded after pre-processing but before analysis due to inadequate registration of the subject MRI data.

### Neuroimaging

T1-weighted images were obtained on a 3.0 T Philips Achieva scanner with voxel size 1.0 x 1.0 x 1.0 mm^3^ with parameters: TR 9.9 ms; TE 4.6 ms; 240 x 231 matrix; FOV 24 cm.

Diffusion Weighted Images (DWI) were acquired using a DWI sequence totaling 11 min, with parameters: 70 axial slices (2 mm thick), FOV = 256 mm x 256 mm x 140 mm, TR/TE 8,657/86 ms, no gap, with a 128x126 acquisition matrix, 28 gradient images collected with b-value = 1,500 s/mm^2^.

### Registration and sampling

T1 and DWI data were visually inspected for major artifacts and signal drop off. T1 images were bias field corrected using ANTs (Avants et al., 2009), N4 BFC (Tustison et al., 2010) tool, and manually skull-stripped in Brainsuite16 (Shattuck et al., 2001). DW images were first visually inspected for motion artifact, and noisy volumes were excluded. DW images were processed through FSL's eddy (Andersson and Sotiropoulos, 2016) for eddy current correction and subject motion correction. DW images were skull-stripped using DSI Studio followed by tensor estimation using FSL's DTIFIT. Tensors were then formatted for use with TSA with DTI-TK's toolbox. The generation of a dataset-specific template to create the medial representations of white matter tracts created inconsistent registration results, and thus we opted to use the adult template publicly available on the DTI-TK site as the registration target. We sequentially registered all subjects through a rigid, affine, then non-linear registration process through the DTI-TK toolbox to align subjects into the atlas space (Zhang et al., 2007). The unique registration protocol within the TSA approach first registers the 6 tensors from each subject's full DTI data to the 6 tensors of the template atlas space. Then, the inverse transform is applied to the previously annotated atlas to then transform the delineated WM tracts back into the patient space. Each resulting registration was manually reviewed for misregistration, especially along the outer boundaries of WM tracts. We used the same toolbox to generate fractional anisotropy (FA) values from the registered subject DT images. Mean diffusivity (MD) values were also calculated and analyzed, but no significant results were found. The FA values within the boundaries of a WM tract were then projected onto a thinner, sheet-like representation of the tract, which snakes through the tract's mid-plane. Each point on that medially located surface held the average value of the FA values projected onto it.

Eleven major white matter tracts available in the standard release of the software were tested: the corpus callosum (CC), and bilateral cortico-spinal tracts (CST), inferior fronto-occipital fasciculi (IFOF), inferior longitudinal fasciculi (ILF), superior longitudinal fasciculi (SLF), and uncinate fasciculi (UF).

### Analysis

A supra-threshold statistical model was used to assess differences in FA between patient groups and healthy controls (Yushkevich et al., 2009). At each point on the medial surface of the tract, a two-sample *t*-test was computed. An arbitrary value t_0_ was used to extract clusters on the medial surface for which their t values are less than t_0_. The size of the cluster (in terms of number of points of the surface) was then collected into a histogram. This process was repeated 10,000 times, but for each instance, the labels of the subjects were randomly permuted. Thus, a non-parametric permutation-based cluster analysis method (Nichols and Holmes, 2002) was used to correct for the family-wise error rate (FWER), considering the number of WM tracts. The threshold *p*-value was set to 0.01 and the number of permutations to 10,000. We included age at study in the general linear model in the TSA pipeline to control for relevant confounding factors. For quality assurance, all corrected clusters were overlaid across each subject's individual FA map. If the cluster aligned outside of or along the edge of the white matter skeleton for the majority of patients, then the cluster would be omitted as we could not determine if the results were due to inadequate registration.

Visualizations of the statistically significant clusters as determined after multiple comparisons correction were created in ParaView (Ahrens et al., 2005), an open-source data analysis and visualization application.

## Results

Demographics for the three comparison groups are shown in Table 1. The majority of children in the surgery group had a diagnosis of pilocytic astrocytoma, while those in the surgery and chemotherapy group were younger at diagnosis, were most often diagnosed with medulloblastoma, and had been off treatment longer.

### Tract specific analysis

**Figure 1:**
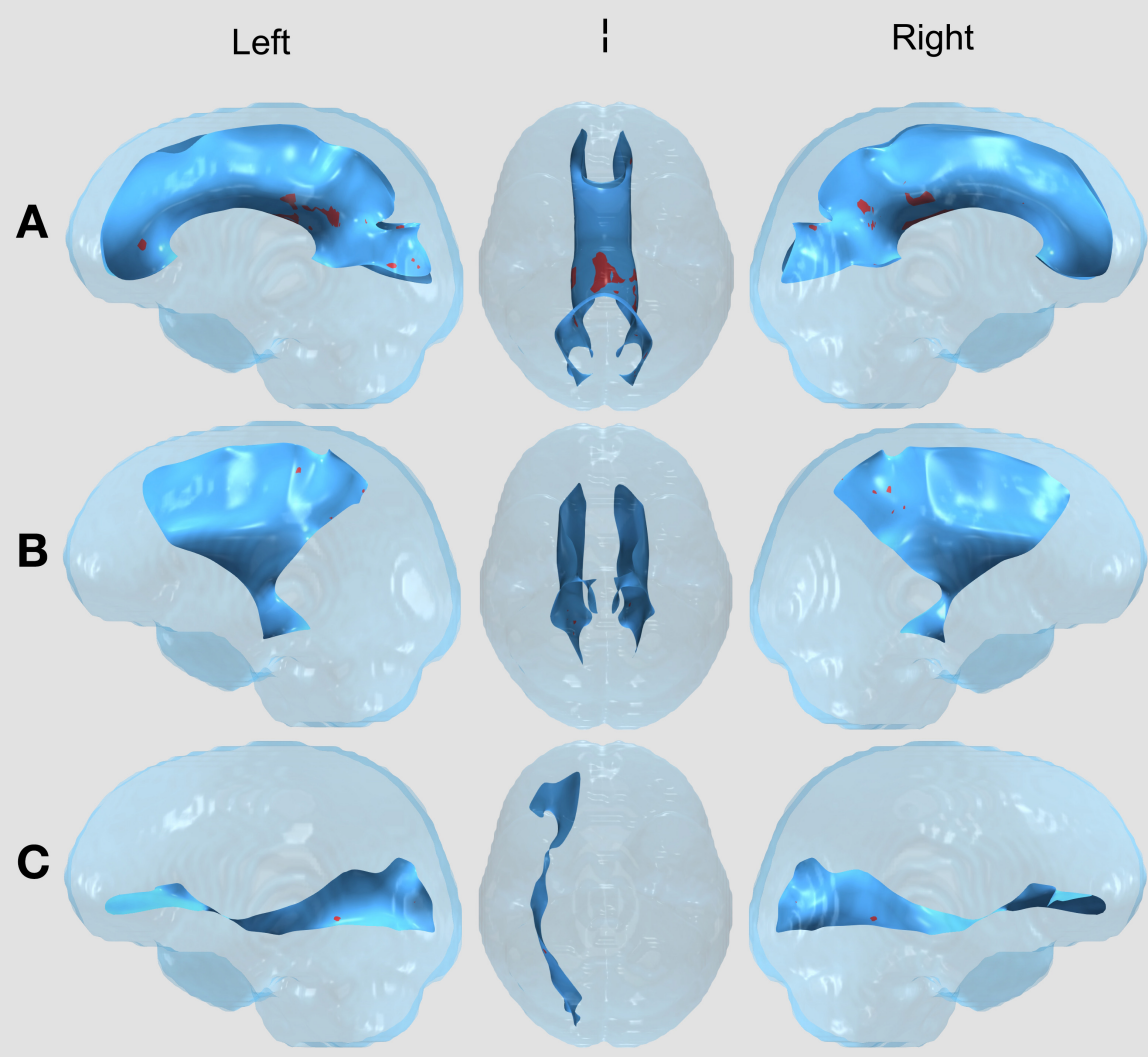
WM Tract Results in the Surgery vs. Healthy Control comparison. All results displayed on a transparent glass brain. The background tract is in blue while the clusters after multiple comparisons correction are displayed in red. The three images, left to right, display results in a leftward facing sagittal view, inferior facing axial view, and rightward facing sagittal view in the **(A)** Corpus callosum (CC); **(B)** Bilateral corticospinal tract (CST); and **(C)** Right inferior frontal occipital fasciculus (right IFOF).

**Figure 2:**
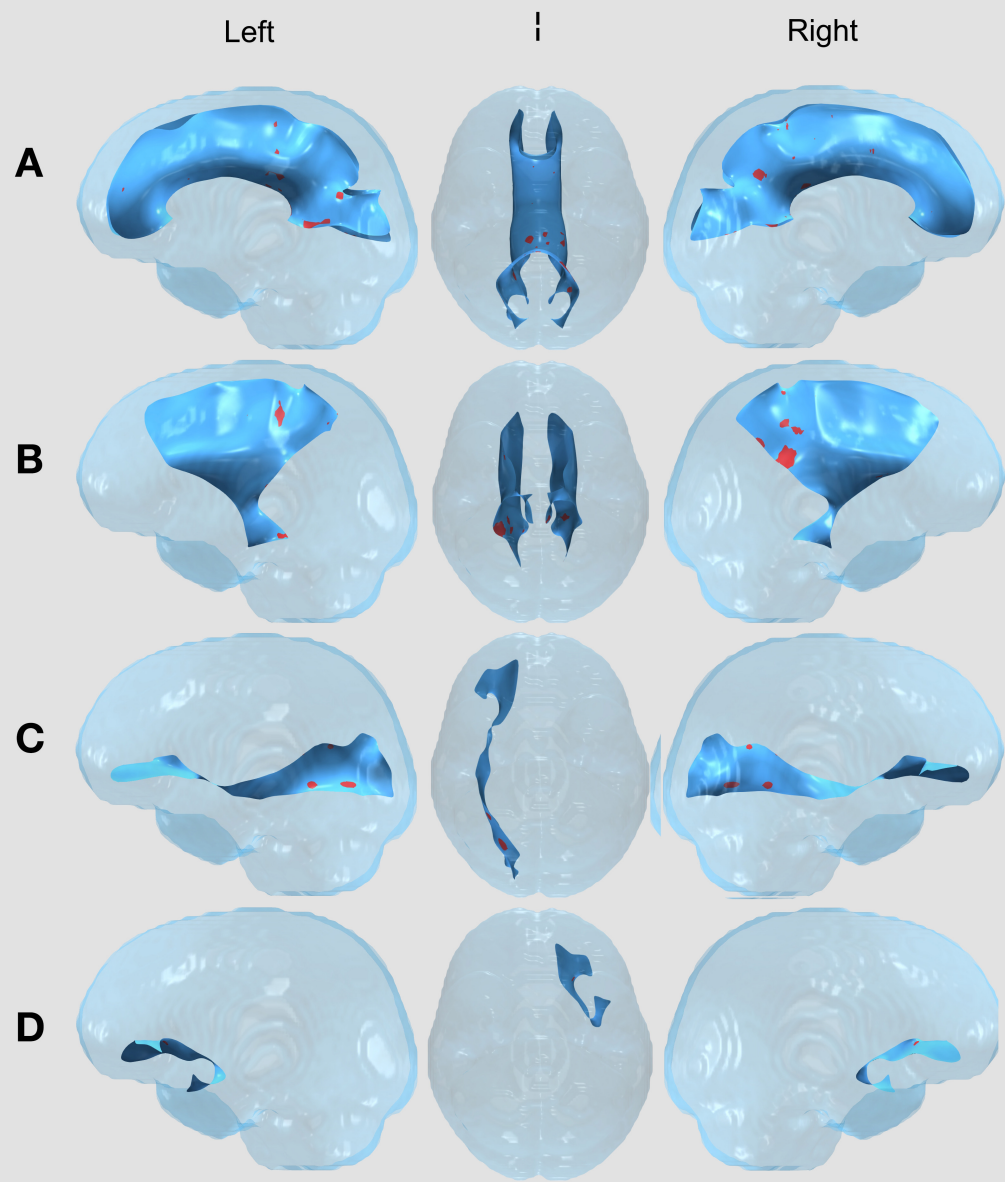
WM Tract Results in the Surgery and Chemotherapy vs. Healthy Controls comparison. All results displayed on a transparent glass brain. The background tract is in blue while the clusters after multiple comparisons correction are displayed in red. The three columns, left to right, display results in a leftward facing sagittal view, inferior facing axial view, and rightward facing sagittal view in the **(A)** Corpus callosum (CC); **(B)** Bilateral corticospinal tract (CST); **(C)** Right inferior frontal occipital fasciculus (right IFOF); and **(D)** left uncinate fasciculus (left UF).

Between the two treatment groups of children with brain tumors (surgery vs. surgery and chemotherapy), there were no significant FA differences in any of the 11 tracts after correcting for multiple comparisons using the permutation-based cluster analysis. However, we found statistically significant group differences in four tracts (the CC, left and right CST, and right IFOF) between the surgery group and healthy controls (Figure 1) and in five tracts (the CC, left and right CST, right IFOF, and left UF) between the surgery and chemotherapy group and controls (Figure 2). In each group comparison, the red clusters indicate areas of lower FA in the patient population in comparison to healthy controls. There were no clusters which indicated higher FA in the patient population compared to healthy controls. Extra figures which provide zoomed in images of the largest clusters along with t-statistic color maps are available as Supplementary material online.

### Surgery vs. healthy controls

In the CC, Figure 1A, we see a large cluster in the splenium of the CC, indicating lower FA in the surgery group compared to healthy controls. This region contains fibers from the superior temporal, inferior temporal and occipital areas of the brain (Schmahmann et al., 2007). In the bilateral CST, Figure 1B, there are small clusters toward the posterior part of the tract. The corticospinal tract connects the motor cortex to the spinal cord through the brainstem and thus is responsible for voluntary movements of the limbs and trunk (Davidoff, 1990). In the right IFOF, Figure 1C, we see clusters in the surgery group in the posterior part of the tract. The IFOF connects all lobes of the brain and is thought to play a key role in non-verbal semantic cognition (Herbet et al., 2017), language and attention (Altieri et al., 2019).

There were also small clusters found in the right inferior longitudinal fasciculus (ILF), left IFOF, and bilateral superior longitudinal fasciculus (SLF). However, these were omitted from visualization and the discussion as the cluster aligned outside of or along the edge of the white matter skeleton for the majority of patients, thus we could not determine if the results were due to misregistration. No clusters were found in the left ILF or bilateral UF.

### Surgery and chemotherapy vs. healthy controls

With visual comparison between Figures 1, 2, there are similar findings between each of the two patient treatment groups and healthy controls in the CC, bilateral CST and R IFOF. Interestingly, there are results in the L UF that exist in the surgery and chemotherapy comparison and not in the surgery comparison. The uncinate fasciculus is a limbic fiber tract which connects the orbitofrontal cortex to the anterior temporal lobes and may affect memory retrieval mechanisms (Olson et al., 2015).

As for the other four tracts, visual inspection between the two comparisons does provide some observable differences in cluster appearance. In the CC, both comparisons have many clusters close to the splenium, but the results in the surgery vs. healthy controls comparison contain a single prominent cluster. In the bilateral CST, both comparisons have clusters in the posterior parts of the bilateral CST. However, the clusters in the surgery and chemotherapy comparison appear larger. In the R IFOF, both comparisons have clusters closer to the posterior parts of the R IFOF, with the surgery and chemotherapy comparison seeming to have larger clusters compared to the surgery comparison. Despite these visual differences between the two patient group comparisons to healthy controls, the direct statistical tests between the surgery and surgery and chemotherapy groups did not contain any clusters.

There were also small clusters found in the bilateral ILF, left IFOF, and bilateral SLF. However, these were omitted from visualization and the discussion as the cluster aligned outside of or along the edge of the white matter skeleton for the majority of patients, thus we could not determine if the results were due to misregistration. No clusters were found in the right UF.

## Discussion

We examined the differences in Fractional Anisotropy (FA) in 11 major WM tracts between two groups of pediatric posterior fossa brain tumor survivors who had received treatment with either surgery alone or surgery + chemotherapy as well as a third group of healthy controls. FA provides some indication of axonal density, organization, and degree of myelination, which in turn may provide information about damage to white matter structures caused by treatment (Rueckriegel et al., 2010). Our results were calculated using a medial representation of several tracts and a deformable shape analysis technique, Tract Specific Analysis (TSA), which projects areas of significant WM differences between groups onto surfaces.

There were several clusters found in the comparisons between the children treated with surgery and healthy controls, and between the children treated with surgery and chemotherapy and healthy controls, even after regressing out the age covariate. Both sets of comparisons demonstrate significantly lower FA in patients in the corpus callosum, bilateral CST, and right IFOF, than in healthy controls. Comparing the surgery and chemotherapy group to controls demonstrated an additional cluster of lower FA in the left UF.

Our findings of lower FA in children with posterior fossa brain tumors in the CC and CST support those found in previous studies of pediatric brain tumor survivors. In a longitudinal study of pediatric brain tumor patients from baseline after surgery through treatment to 36 months later, Glass et al. (2017) utilized TBSS and reported reduced FA values in the CC and CST of patients. However, this investigation studied children treated with a combination of surgery, chemotherapy, and radiation therapy. Our study shows similar findings of lower FA in these tracts in both patient groups, indicating that WM damage to these tracts may be attributed to the presence of a posterior fossa mass, resulting in increased intracranial pressure and hydrocephalus, in combination with surgical resection rather than to cranial irradiation. Forty percent of children in the surgery and chemotherapy group had moderate hydrocephalus noted on MRI or CT by neuroradiologists at diagnosis as did 27% of children in the surgery treatment group. Expanded ventricular size causes stretching and compression of white matter tracts, resulting in axonal and blood vessel injury (Del Bigio, 2001). Acute hydrocephalus was shown to decrease FA in the corpus callosum, a finding that did not return to normal values after insertion of a shunt and relieving the pressure (Assaf et al., 2006). A study of children with posterior fossa tumors treated with surgery found reduced FA in the cerebellum, callosal body, corona radiata and frontal cortex (Rueckriegel et al., 2010). Since lower FA was present in both patient groups, we are unable to ascribe such changes to either surgery or to chemotherapy, or even to the effects of the tumor alone.

Notably, a study of children with bone and soft tissue tumors outside the CNS who received chemotherapy with various agents, some of the same used in our patients (most often cisplatin and cyclophosphamide), also reports lower FA in the CC and CST after treatment (Sleurs et al., 2018). The chemotherapy agents used to treat patients in this study have neurotoxic effects, the cellular basis for which is difficult to specify in humans. For example, *in vitro*, cisplatin is more toxic to oligodendrocytes than to rapidly dividing cancer cells (Dietrich et al., 2006). In an animal model, cisplatin, thiotepa, and cyclophosphamide had a dose-dependent neurotoxic effect that included dendritic swelling in the thalamus, dentate gyrus, caudate nucleus and cortex (Rzeski et al., 2004). Such effects at the cellular level could at least partially explain the microstructural brain tissue changes occurring after treatment in many pediatric brain tumor studies.

White matter loss or damage can have lasting effects on learning and cognition. The CC plays a critical role in processing motor, sensory, and cognitive signals from both hemispheres. Palmer et al. (2012) and Aukema et al. (2009) found FA in the CC to be positively associated with processing speed in survivors of pediatric brain tumors who had been treated with surgery, chemotherapy, and radiation. Although research linking reduced FA in the CST to motor deficits in pediatric brain tumor populations is sparse, studies have found an initial decline in FA in the CST in 3 children after brain tumor treatment (Hua et al., 2012), and in children after proton beam irradiation (Uh et al., 2015) that recovered over time. The IFOF is an association fiber system which connects the occipital cortex, temporo-basal areas and superior parietal lobe to the frontal lobe and may play a role in reading, attention, and visual processing (Wu et al., 2016). In studies of pediatric brain tumor survivors who received varying levels of treatment with surgery, chemotherapy, and radiation, Aleksonis et al. (2021) and Aukema et al. (2009) reported lower mean FA in the right IFOF with Aukema et al. finding a positive correlation between mean FA and processing speed. In another study of pediatric survivors of brain tumors treated with all three treatment modalities, lower mean FA was reported in the bilateral UF as well (Riggs et al., 2014).

To explore the laterality of findings with reduced FA in the left UF in the surgery and chemotherapy group and in the right IFOF in both patient groups, we first looked at tumor location as a possible factor. However, half of children in the surgery and chemotherapy group had midline tumors arising from the fourth ventricle, while 30% were located in the right cerebellum and 20% in the left. When combining patient groups, location was evenly distributed, with 40% of children having tumors arising from the fourth ventricle or cerebellar vermis, 36% in the right cerebellum, and 24% in the left cerebellum. The lack of a significant number of children with lateral tumor location diminishes the likelihood of location as a factor. The area of reduced FA in the right IFOF shown in Figure 2C is in the most posterior region of the fasciculus and could have been very close to the site of tumor resection. Surgical approach data were not collected as part of this study, and it remains unclear why damage to that tract would be unilateral.

Resection of a posterior fossa tumor results in reduced FA in supratentorial WM tracts of the cerebello-thalamo-cortical pathway, parts of which are quite distant from the tumor site (Gomes et al., 2021). Law et al. (2015) studied children with medulloblastoma treated with surgery, chemotherapy and cranial irradiation where over 90% of tumors were located in the midline. They also concluded that the cerebrocerebellar pathways showed evidence of injury, more so on the left than the right, and theorized that a right-to-left gradient of brain maturation could explain greater vulnerability of the left hemisphere (Kucyi et al., 2012; Law et al., 2015). This, along with the fact that our patients treated with surgery and chemotherapy were significantly younger at diagnosis than those treated with surgery, may support the finding of reduced FA in the left UF in the group of children who had a neurotoxic insult earlier in life.

The critical difference between most studies reporting on WM microstructure in this population and our study is that we stratified our population according to treatment type to understand the effect that different treatments have on the recovering and developing pediatric brain. While the studies cited above examined the effects of all treatments and found lower FA in the CC, CST, and IFOF, our study examined children treated with surgery only and found similar results in all three WM structures. These results may indicate lasting effects on the supratentorial brain by infratentorial tumors or resection that may be exacerbated by or at least persist through adjuvant treatment as evidenced by our similar findings in this study's surgery and chemotherapy group and from other studies on patient groups who had also been treated with cranial irradiation. Supratentorial WM structures with projections to the cerebellum, such as the CST, may suffer axonal degeneration if the tumor or surgical resection damages these cerebellar extensions. Our lack of findings in the direct comparison between the two patient groups makes it difficult to parse out the additive effect of chemotherapy to surgical resection. However, our findings in the UF in the surgery and chemotherapy vs. healthy control comparison may indicate a relationship between chemotherapeutic agents and damage to the UF because there are no similar significant findings in the comparison of children treated with surgery to controls.

To reduce the regions of interest to 11 specific white matter tracts, the continuous medial representations of the WM tracts do not capture more distal neuronal extensions. Thus, TSA is unable to reveal full WM connections, especially in deeper WM structures. Additionally, the diffusion tensor model is inherently unable to accurately describe voxels which contain WM fibers that may cross, fan, bend, or branch and may generate underestimated FA values in those regions (Seunarine and Alexander, 2014). One recognized limitation of FA is the inability to account for multiple fiber populations which thus limits our ability to relate differences in observed diffusion to WM characteristics such as myelination and fiber density (Beaulieu, 2002). Thus, our future research will utilize methods which can distinguish multiple fibers (Garyfallidis et al., 2014). Due to our usage of the adult template during the registration process, it is possible that some individual subject registration results may be skewed as the distance from subject space to template space is developmentally larger. However, each resulting registration was manually reviewed for misregistration, especially along the outer boundaries of WM tracts. Clusters which fell along areas which were more prone to misregistration were not included in the analysis. Additionally, the smaller N may relate to our lack of findings between the surgery and surgery and chemotherapy group as it is possible that FA differences between the two may be so subtle as to require a larger number of subjects for statistical significance. Our findings in the UF in the surgery and chemotherapy group vs the healthy controls but not in any other comparison may indicate a similar subtlety between surgery and healthy controls and between surgery and surgery and chemotherapy that was not seen in this analysis. To control for the effects of development, we covaried our analysis with age at time of scan. Due to the limitation in the number of patients, we were unable to also control for age at treatment. Finally, patients were not matched to controls by gender, age, language, or handedness, although 48% of controls were siblings of the participants. These limitations as well as the small sample size of this study make conclusive interpretations of the results difficult to produce.

## Conclusion

In conclusion, our study is the first to investigate the impact of chemotherapy and/or surgery separately on microstructural changes in the 11 major WM tracts. Statistically significant tracts with decreased FA in the CC, bilateral CST, and right IFOF were observed in both groups of patients compared to their healthy age-matched sibling controls. Decreased FA was also observed in the UF when comparing the surgery and chemotherapy group to healthy controls. No tracts with significant FA differences were found between the two patient groups. Findings in the surgery vs. control group indicate that surgical resection in the cerebellar region, while necessary, may have effects on the relatively distant supratentorial white matter. Our study findings support others of children with brain tumors that also report decreased mean FA in WM tracts. However, unlike many previous studies, we controlled for tumor location and stratified our dataset by treatment type (Baron Nelson et al., 2021). Further work on this study population will include a third patient group who received surgery, chemotherapy, and radiation in order to further understand WM damage and neurocognitive functioning in pediatric survivors of posterior fossa brain tumors.

## Data availability statement

The datasets presented in this article are not readily available because of privacy or ethical restrictions. Requests to access the datasets should be directed to nlepore@chla.usc.edu.

## Ethics statement

The studies involving human participants were reviewed and approved by Children's Hospital of Los Angeles Institutional Review Board. Written informed consent to participate in this study was provided by the participants' parent/legal guardian.

## Author contributions

MN and NL designed the study. MN analyzed demographic data and JT, NG, and NL analyzed imaging data. JT, SG, and NG refined the analysis protocol. JT developed processing protocols for pediatric MR images, wrote the manuscript, and all authors edited it. All authors contributed to the article and approved the submitted version.

## Funding

This study was funded by NIH grant K23NR014901, Children's Hospital Los Angeles Hemonc/BMT Division Research Funds and supported by The Saban Research Institute (TSRI) of Children's Hospital Los Angeles, awarded to MN. NL is funded by NIH NIBIB grant R01EB025031, NIH NIDCR grant R01DE030286, and TSRI grant 000013228.

## Conflict of interest

The authors declare that the research was conducted in the absence of any commercial or financial relationships that could be construed as a potential conflict of interest.

## Publisher's note

All claims expressed in this article are solely those of the authors and do not necessarily represent those of their affiliated organizations, or those of the publisher, the editors and the reviewers. Any product that may be evaluated in this article, or claim that may be made by its manufacturer, is not guaranteed or endorsed by the publisher.
