# Supplementary material for: White matter tract changes in pediatric posterior fossa brain tumor survivors after surgery and chemotherapy
Source: Front Neuroimaging. 2022 Sep 20;1:845609. doi: 10.3389/fnimg.2022.845609 (PMC10406254; doi:10.3389/fnimg.2022.845609)
Supplement: Supplementary file 1 [file Data_Sheet_1.pdf]

Supplementary Material

Surgery vs Healthy Controls

1 Corpus callosum

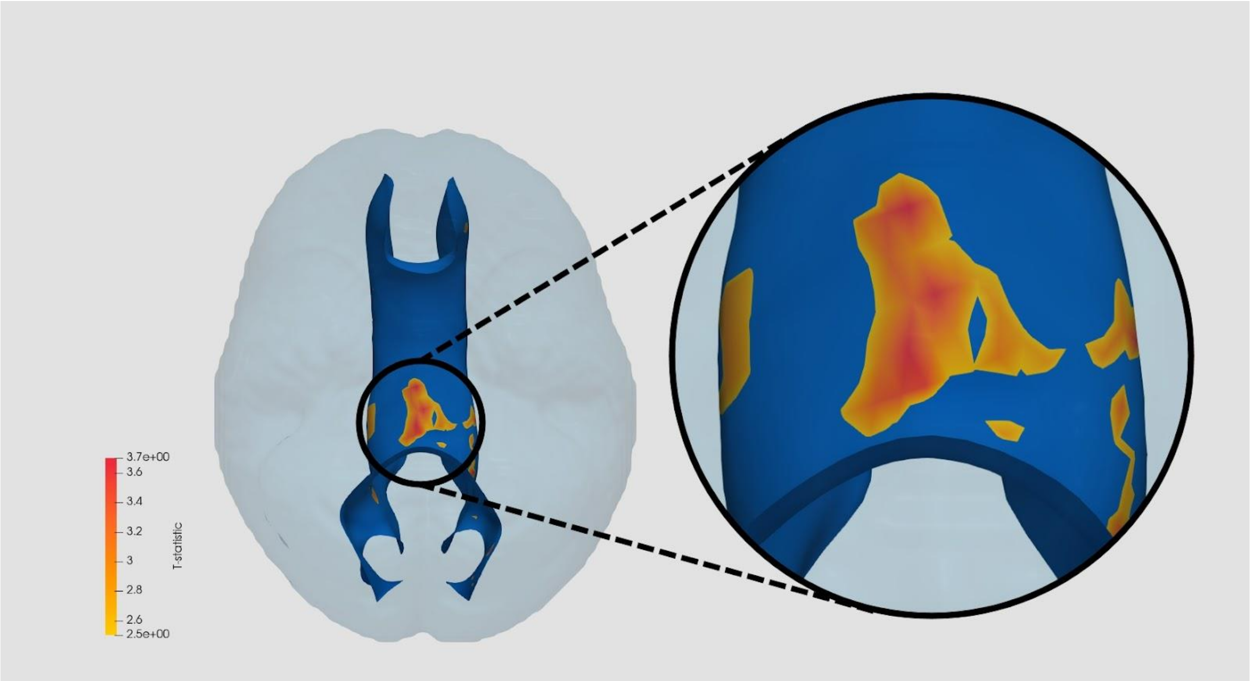

WM Tract Results in the Surgery vs Healthy Controls comparison. All results displayed in a transparent glass brain. The background tract is in blue while the clusters after multiple comparisons correction are displayed in a range from yellow to red depending on their t-value. Displayed here is an inferior facing axial view of the corpus callosum.

2 Left corticospinal tract

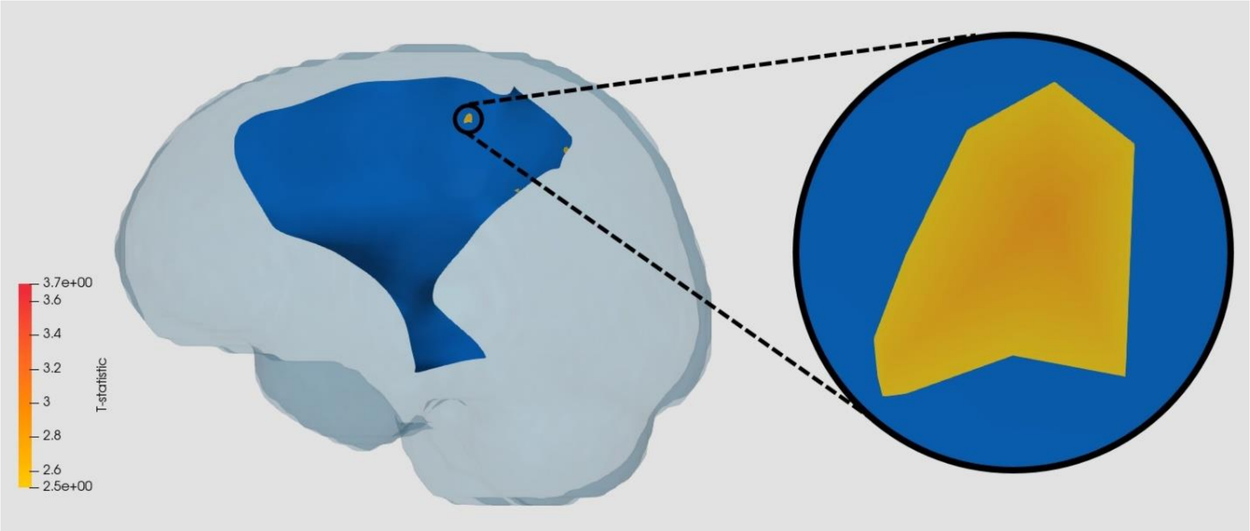

WM Tract Results in the Surgery vs Healthy Controls comparison. All results displayed in a transparent glass brain. The background tract is in blue while the clusters after multiple comparisons correction are displayed in a range from yellow to red depending on their t-value. Displayed here is a leftward facing sagittal view of the left corticospinal tract.

3 Right corticospinal tract

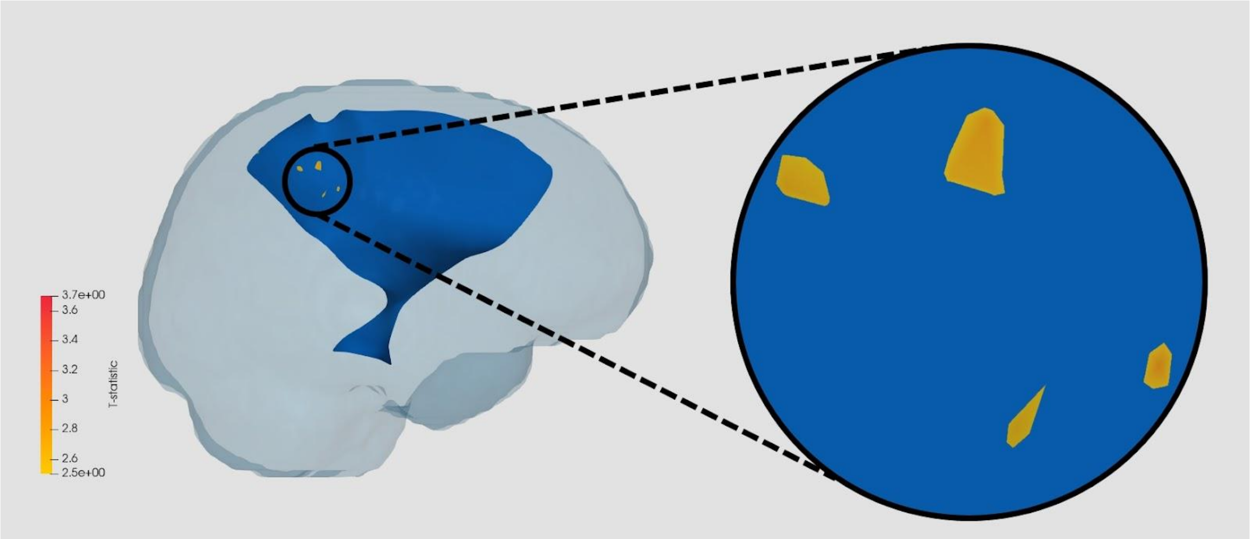

WM Tract Results in the Surgery vs Healthy Controls comparison. All results displayed in a transparent glass brain. The background tract is in blue while the clusters after multiple comparisons correction are displayed in a range from yellow to red depending on their t-value. Displayed here is a rightward facing sagittal view of the right corticospinal tract.

4 Right inferior fronto-occipital fasciculus

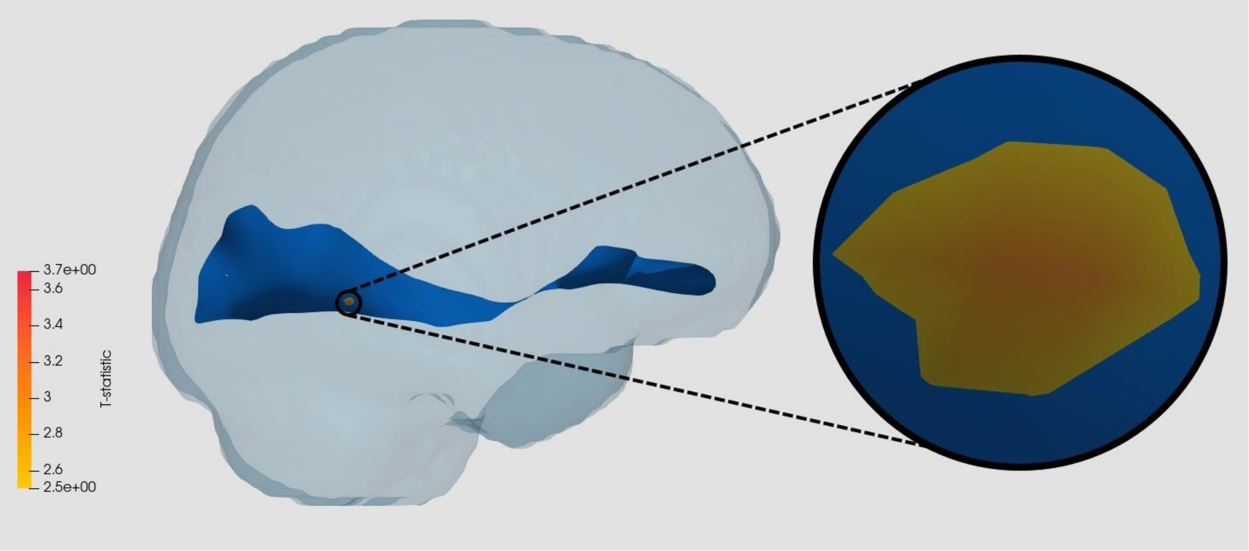

5

WM Tract Results in the Surgery vs Healthy Controls comparison. All results displayed in a transparent glass brain. The background tract is in blue while the clusters after multiple comparisons correction are displayed in a range from yellow to red depending on their t-value. Displayed here is a rightward facing sagittal view of the right inferior fronto-occipital fasciculus.

Surgery and Chemotherapy vs Healthy Controls

6 Corpus callosum

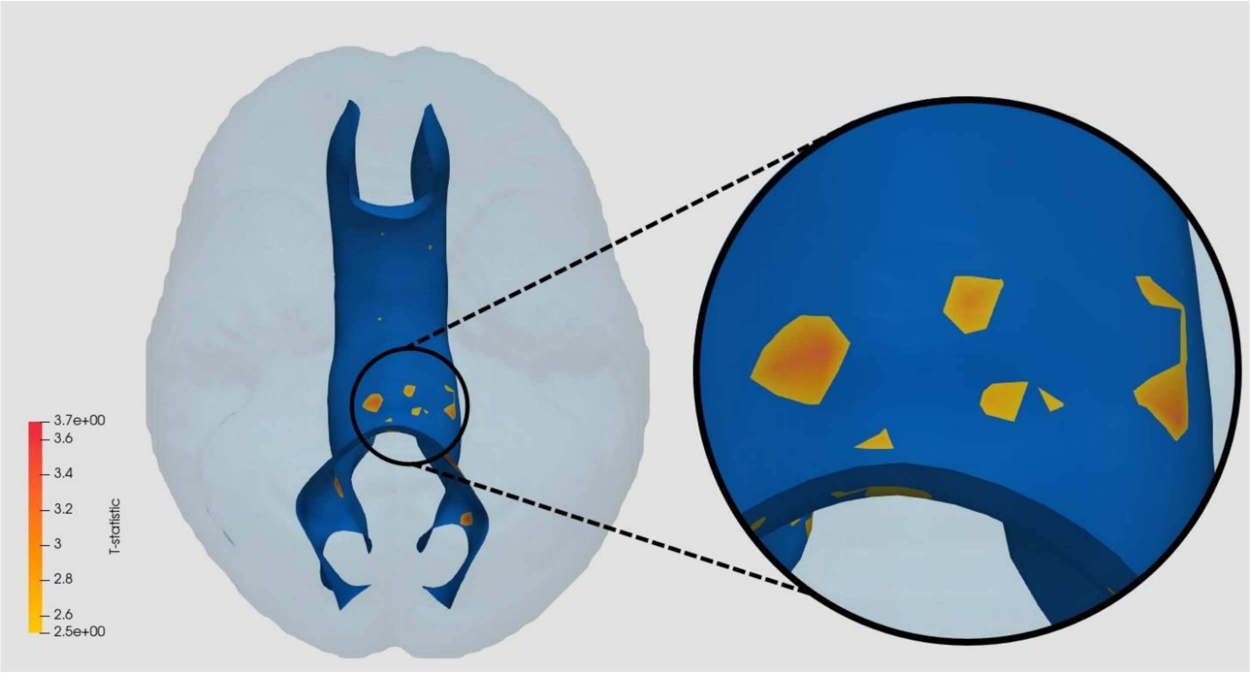

WM Tract Results in the Surgery and Chemotherapy vs Healthy Controls comparison. All results displayed in a transparent glass brain. The background tract is in blue while the clusters after multiple comparisons correction are displayed in a range from yellow to red depending on their t-value. Displayed here is an inferior facing axial view of the corpus callosum.

7 Left corticospinal tract

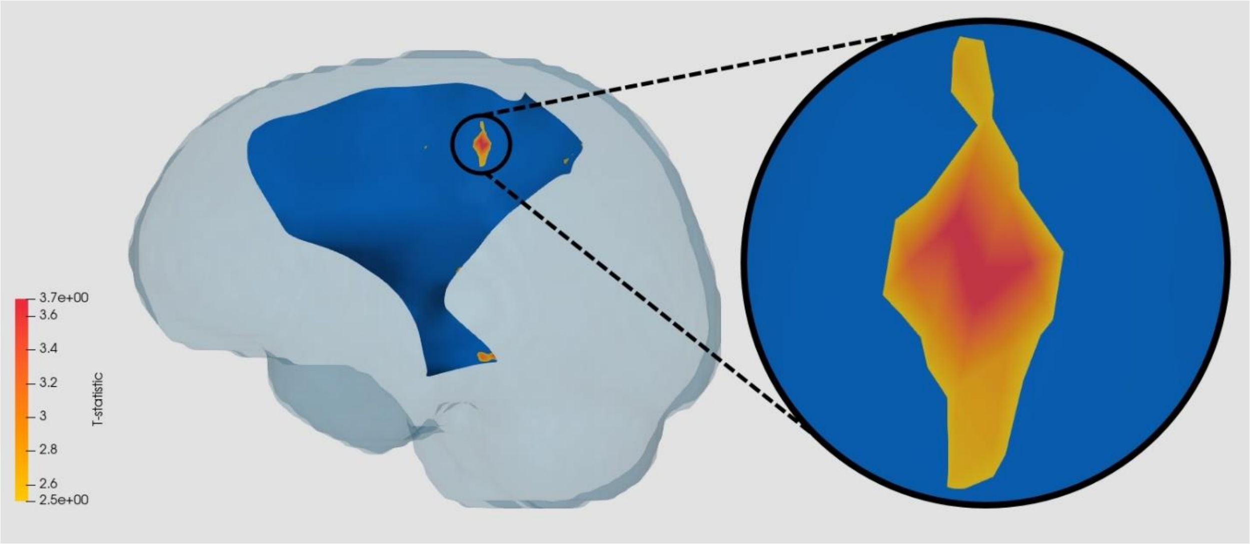

WM Tract Results in the Surgery and Chemotherapy vs Healthy Controls comparison. All results displayed in a transparent glass brain. The background tract is in blue while the clusters after

multiple comparisons correction are displayed in a range from yellow to red depending on their t-value. Displayed here is a leftward facing sagittal view of the left corticospinal tract.

8 Right corticospinal tract

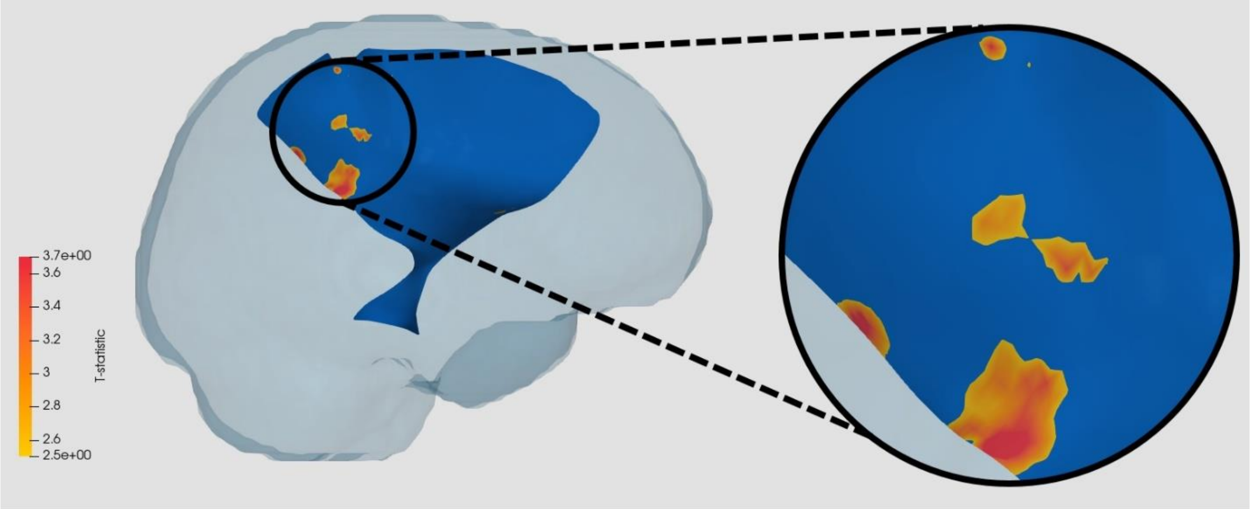

WM Tract Results in the Surgery and Chemotherapy vs Healthy Controls comparison. All results displayed in a transparent glass brain. The background tract is in blue while the clusters after multiple comparisons correction are displayed in a range from yellow to red depending on their t-value. Displayed here is a rightward facing sagittal view of the right corticospinal tract.

9 Right inferior fronto-occipital fasciculus

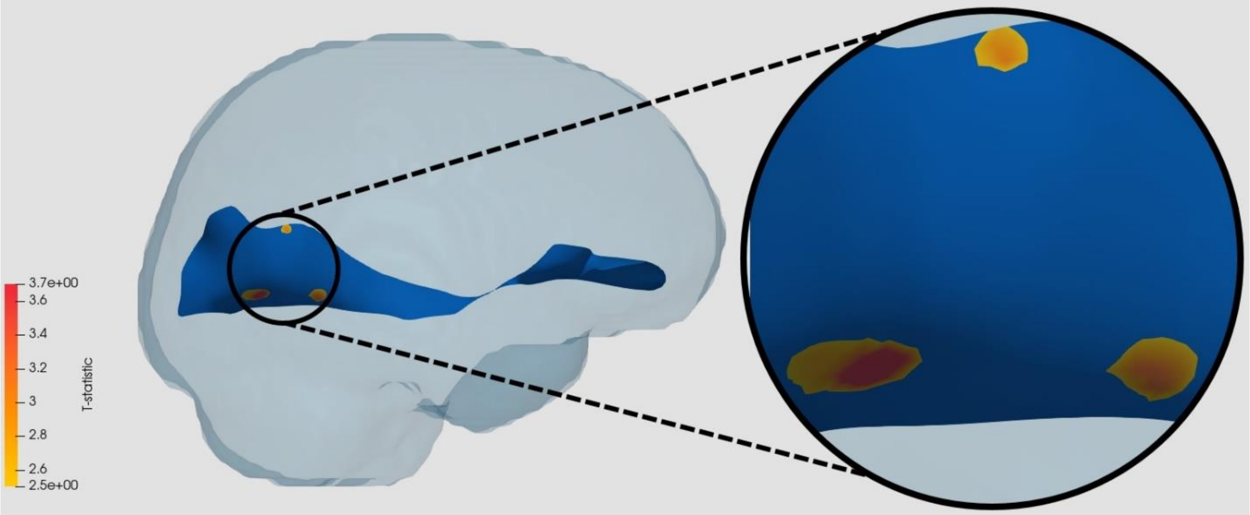

WM Tract Results in the Surgery and Chemotherapy vs Healthy Controls comparison. All results displayed in a transparent glass brain. The background tract is in blue while the clusters after multiple comparisons correction are displayed in a range from yellow to red depending on their t-value. Displayed here is a rightward facing sagittal view of the right inferior fronto-occipital fasciculus.

10 Left uncinate fasciculus

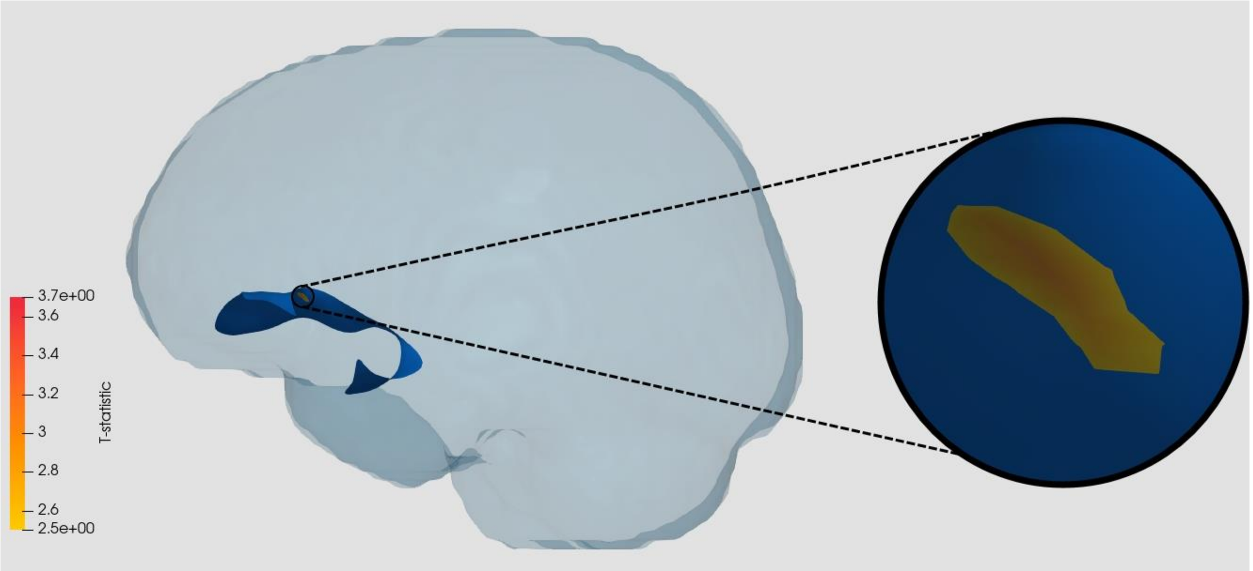

WM Tract Results in the Surgery and Chemotherapy vs Healthy Controls comparison. All results displayed in a transparent glass brain. The background tract is in blue while the clusters after multiple comparisons correction are displayed in a range from yellow to red depending on their t-value. Displayed here is a leftward facing sagittal view of the left uncinate fasciculus
